# Influence of parental structure and chaos on homework anxiety in elementary school students: the mediating role of homework motivation

**DOI:** 10.3389/fpsyg.2024.1399507

**Published:** 2024-10-07

**Authors:** Jing Li, Yadong Ding

**Affiliations:** School of Public Policy and Management, China University of Mining and Technology, Xuzhou, Jiangsu, China

**Keywords:** parental structure, parental chaos, homework anxiety, autonomous motivation, controlled motivation

## Abstract

The present study aimed to examine the relationship between parental structure, parental chaos, and homework anxiety among elementary school students, as well as the mediating roles of autonomous and controlled motivation in relation to homework. A total of 1,118 elementary school students from grades 4 to 6 completed the Parents as Social Context Questionnaire, Homework Anxiety Scale, and Homework Motivation Scale. Results indicated that parental structure was negatively associated with homework anxiety in elementary school students, while parental chaos exhibited a positive correlation with such anxiety; furthermore, both homework autonomous and controlled motivation mediated the relationship between parental structure and chaos with homework anxiety. Specifically, a structured parenting environment could mitigate homework anxiety by enhancing students’ autonomous motivation; conversely, chaotic parenting environments were associated with increased homework anxiety through heightened controlled motivation while simultaneously diminishing autonomous motivation. These findings offer novel insights into alleviating homework-related anxiety among elementary school students and provide empirical support for developing educational interventions focused on parenting practices.

## Introduction

Homework is defined as tasks assigned by teachers for completion outside of regular school hours (Cooper, 1989), intended to reinforce classroom learning which fosters self-regulation and academic growth (Moè et al., 2018). However, media reports alongside direct observations reveal that many elementary school students are often accompanied by negative emotional responses in their homework. Prior research has demonstrated that these students experience lower levels of positive emotions (e.g., pleasure) coupled with elevated levels of negative emotions (e.g., anxiety) during homework compared to other school activities (Dettmers et al., 2011; Goetz et al., 2012). In China’s competitive educational landscape—where success is closely tied to academic achievement—students face significant pressure regarding advancement opportunities toward higher education (Liu et al., 2019; Kipnis, 2011). Consequently, amidst this intense competition surrounding academics where extracurricular learning heavily relies on completing assignments at home—a scenario frequently linked with heightened students’ anxiety (Katz et al., 2012; Pomerantz et al., 2007). Students may exhibit maladaptive behaviors including reduced effort investment or increased procrastination leading ultimately towards disengagement or withdrawal from studies altogether (Katz et al., 2014), underscoring an urgent need for exploration into factors contributing to this phenomenon known as “homework anxiety.”

Existing literature highlights substantial adverse effects stemming from prolonged experiences of such anxieties—including undermining perseverance when faced with challenging tasks or fostering avoidance tendencies (Pekrun et al., 2011). Moreover, long-term experiencing anxiety can precipitate severe outcomes like suicidal ideation or dropout (Pekrun et al., 2002). According to broaden-and-build theory concerning emotional states repeated encounters involving negativity may entrap learners within detrimental cycles (Fredrickson, 2004). For instance, persistent anxiety related to daily homework might engender mental distress, constrain cognitive flexibility, limit resource utilization, leading to diminished confidence regarding one’s capabilities and further exacerbated anxiety, creating a vicious cycle.

For primary school students, homework is typically required to be completed at home, and the impact of the parenting environment and parental behaviors on their homework anxiety is undoubtedly significant and direct. As crucial supporters in their children’s education, it is essential for parents to cultivate a positive parenting environment and provide appropriate guidance. The concepts of parental structure and chaos represent distinct types of parenting environments wherein parents manage their children’s behavior during interactions; these have a pronounced effect on elementary students’ homework anxiety. Parental structure pertains to the degree to which parents furnish their children with information regarding pathways toward achieving desired outcomes while offering support and guidance along those paths (Farkas and Grolnick, 2010). This primarily manifests through clear, explicit, and detailed guidelines, expectations, rules provided by parents (Flamm and Grolnick, 2013; Skinner et al., 2005). In a structured parenting environment, parental behaviors are characterized as consistent, stable, predictable, and reliable. Conversely, parental chaos refers to situations where parents obscure or interfere with relevant information concerning their children’s pathways toward achieving desired outcomes—typically manifesting as disorganized or undisciplined family dynamics that include inconsistent or arbitrary parenting practices (Flamm and Grolnick, 2013; Skinner et al., 2005). Established research indicates that structured parenting environments foster intrinsic motivation in children while guiding them towards self-regulation, enhance feelings of control and competence, encourage positive engagement in learning activities while simultaneously reducing anxiety levels, whereas chaotic family settings yield opposite effects (Farkas and Grolnick, 2010; Griffith and Grolnick, 2014; Grolnick et al., 2015; Mouratidis et al., 2018; Skinner et al., 2005). Given the pivotal role played by parental structure versus chaos in influencing elementary school students’ homework anxiety levels necessitates further exploration into this relationship to elucidate how these factors intrinsically affect such anxieties.

### Parental structure and chaos and homework anxiety

Parental structure significantly contributes to the healthy development of elementary school students. When completing homework within a structured environment established by parents who set clear expectations alongside reasonable goals for their children—allowing them autonomy over planning actions—their understanding correlates behavior with important outcomes such as success or failure (Skinner et al., 1990). Consequently, children perceiving tasks as manageable will likely experience more favorable emotions leading to reduced anxiety surrounding homework completion. Conversely, in chaotic environments, elementary-aged learners may feel diminished control over critical outcomes during task execution, leading them to perceive themselves as less integral participants in processes affecting results—which can engender low feelings of competence coupled with frustration regarding unmet needs for achievement (Griffith and Grolnick, 2014; Farkas and Grolnick, 2010; Flamm and Grolnick, 2013), resulting ultimately in loneliness, failure, and self-doubt (Chen et al., 2015), thereby escalating overall anxiety levels. Previous studies have examined various aspects related specifically towards different styles like autonomy support impacting student emotional responses during assignments (Luo et al., 2016; Moè et al., 2018; Valdés-Cuervo et al., 2022); however, to date, no investigations have explored the influence of parental structure and chaos on homework anxiety. Given the strong association between structure, chaos and elementary school homework anxiety, the present study hypothesized a negative association between parental structure and homework anxiety, and a positive association between parental chaos and homework anxiety in elementary school students.

### Parental structure and chaos, homework motivation, and homework anxiety

According to Self-Determination Theory (SDT), parental structure and chaos can significantly influence various dimensions of students’ development by affecting their motivation to learn (Ryan and Deci, 2000; Vansteenkiste et al., 2012). Parental structure is positively correlated with the enhancement of students’ intrinsic motivation. Parents establish behavioral boundaries for their children through effective communication, setting clear and explicit rules and expectations, and consistently enforcing these guidelines (Farkas and Grolnick, 2010; Grolnick et al., 2014; Grolnick et al., 2015). Initially subjected to external pressures—such as adherence to rules—students gradually internalize these behaviors through parental modeling, direct instruction, and support in rule enforcement. This process fosters a transition from externally driven behavior to more autonomously regulated actions, thereby enhancing autonomous motivation while diminishing controlled motivation (Deci and Ryan, 2012; Grolnick et al., 2015). In contrast, parents operating within chaotic environments often struggle with clarity in expectations and guidance due to their own confusion or ambivalence. Such parents may focus excessively on demanding outcomes without providing coherent strategies for navigating academic tasks (Skinner et al., 2005), which can hinder the development of autonomous regulation in their children and potentially decrease autonomous motivation while increasing controlled motivation.

Autonomous motivation pertains to learning driven by personal interest or perceived value of the material learned; conversely, controlled motivation involves engagement primarily aimed at avoiding pressure from both internal and external sources (Deci and Ryan, 2000). Students exhibiting higher levels of autonomous motivation tend to engage deeply with learning activities out of genuine interest rather than obligation. Consequently, they experience more positive emotions and reduced anxiety when completing homework assignments (Liu et al., 2019; Ketonen et al., 2018). Conversely, those motivated by external pressures may show less engagement with homework tasks. Empirical studies have demonstrated that autonomous motivation correlates positively with numerous beneficial educational outcomes—including academic engagement, effortful study habits, creative thinking abilities—and favorable emotional experiences related to academics as well as overall performance (Datu, 2017; Guo, 2018; Liu et al., 2013; Mouratidis et al., 2018; Ketonen et al., 2018). On the other hand, controlled motivation has been linked with adverse educational consequences such as heightened academic anxiety, boredom, procrastination, and poorer performance (Mouratidis et al., 2018; Ketonen et al., 2018; Wijsman et al., 2018). Thus, the present study hypothesized that homework motivation (including autonomous and controlled motivation) may mediate the relationship between parental structure, chaos and homework anxiety in elementary school students.

### The current research

Despite the abundance of research on homework anxiety among students, particularly in the context of policies aimed at alleviating academic pressures, this issue has not received the necessary attention it deserves. A thorough assessment of this aspect has the potential to significantly expand our empirical understanding and illuminate the fundamental causes of students’ homework anxiety. Furthermore, the intricate interplay between parental structure and chaos, along with homework motivation, and their subsequent impact on anxiety levels, remains under-explored and under-emphasized. By establishing correlations between these factors, it is possible to provide new perspectives and bases for the alleviation of elementary school students’ homework anxiety, as well as provide targeted suggestions and theoretical support for the development and improvement of parent parenting-related interventions in educational practice.

**Figure 1:**
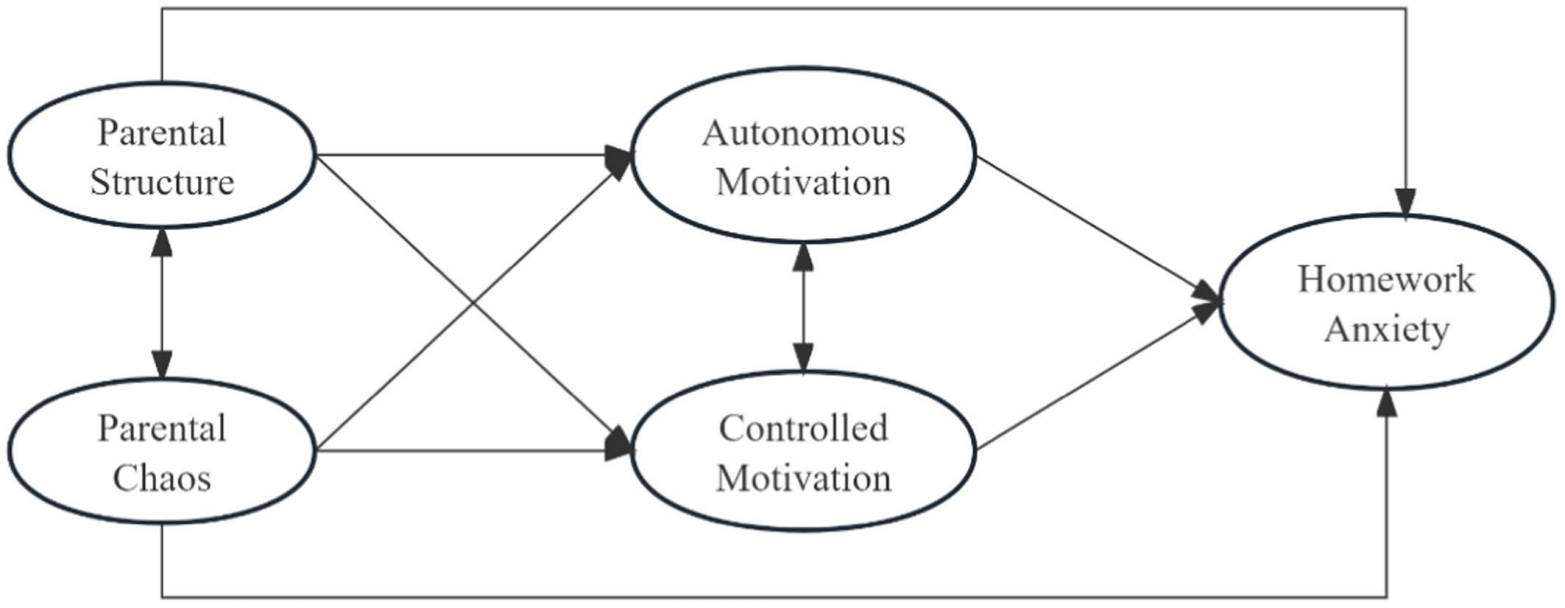
The hypothetical model.

To summarize, this study comprehensively explores the relationship between parental structure, chaos, and homework anxiety among elementary school students, as well as the mechanism of homework motivation, which is conducive to a deeper and more systematic understanding of how these factors influence homework anxiety. Building on existing research, the present study proposes the following hypotheses: (1) a negative correlation exists between parental structure and homework anxiety, while a positive correlation is observed between parental chaos and homework anxiety in elementary school students; (2) both autonomous motivation and controlled motivation mediate the relationship between parental structure, chaos, and homework anxiety in this demographic. The hypothesized model is shown in Figure 1.

## Methods

### Participants and procedure

Students in grades 4 to 6 from two primary schools in Hebei Province, China, were selected as the survey objects, employing a class-based cluster sampling method. A total of 1,156 questionnaires were distributed, yielding 1,118 valid responses and a recovery rate of 96.71%. The participants’ ages ranged from 9 to 13 years old (*M* = 10.56, *SD* = 1.735), with a distribution comprising 623 (55.72%) in the fourth grade, 272 (24.33%) in the fifth grade, and 223 (19.95%) in the sixth grade; among them were 573 (51.25%) boys and 545 (48.75%) girls. Data collection was conducted during the middle of the fall semester. Prior to initiating the formal survey process, researchers informed participants that their involvement was voluntary and that they could withdraw at any time without repercussions. Classroom teachers communicated the study’s purpose to parents, securing informed consent from all guardians involved with the subjects’ participation. The formal surveys took place within quiet classroom settings under the supervision of professionally trained graduate student researchers who provided instructions while collaborating closely with classroom teachers.

### Measure

#### Parental structure and chaos

The Parents as Social Context Questionnaire developed by Skinner et al. (2005) were used to assess parental structure and chaos experienced by students; responses were recorded on a 5-point scale ranging from 1 “strongly disagree” to 5 “strongly agree.” Parental structure comprised four items (e.g., “My parents explain the reasons for our family rules.”). In the preset study, the internal reliability for parental structure was 0.809. Confirmatory factor analysis indicated a good model fit: *χ^2^/df* = 4.622, RMSEA = 0.056, CFI = 0.996, TLI = 0.987, and SRMR = 0.012. Parental chaos consisted of 4 items (e.g., “My parents keep changing the rules on me.”). In the present study, the internal reliability for parental chaos was 0.778. Confirmatory factor analysis indicated a good model fit: *χ^2^/df* = 0.134, RMSEA = 0.000, CFI = 1.000, TLI = 1.004, and SRMR = 0.001. Mean scores across these four items served as individual scores reflecting either parental structure or chaos—higher scores denoting greater levels of each construct.

#### Homework motivation

The Homework Motivation Scale, developed by Katz et al. (2011), was employed to measure students’ motivation for completing homework. The scale encompasses two dimensions: autonomous motivation (11 items, e.g., “I do homework because it is interesting to me.”) and controlled motivation (8 items, e.g., “I do my homework because if I do not I would feel bad when I meet the teacher.”). A 5-point scale was used, with 1 indicating “not at all” and 5 indicating “very much.” Mean scores of the 11 items were calculated as the individual scores of the autonomous motivation, where higher scores reflect greater levels of autonomous motivation. In this study, the coefficient of internal consistency for autonomous motivation was 0.936. Confirmatory factor analysis of autonomous motivation indicated an acceptable model fit: *χ^2^/df* = 8.869, RMSEA = 0.083, CFI = 0.976, TLI = 0.956, and SRMR = 0.030. One item from controlled motivation scale that exhibited low and nonsignificant factor loading was excluded from the subsequent analyses in the present study; thus, the mean score of the remaining seven items served as each individual’s score for controlled motivation—higher scores indicating increased levels of controlled motivation In the present study, the internal consistency coefficient for controlled motivation scale was 0.860. The model fitness of controlled motivation generated by confirmatory factor analysis was acceptable: *χ^2^/df* = 8.727, RMSEA = 0.082, CFI = 0.976, TLI = 0.954, SRMR = 0.034.

#### Homework anxiety

Students’ homework anxiety was measured using the anxiety subscale of the Homework Emotions Questionnaire adapted by Goetz et al. (2012). This subscale comprised four items, such as “I feel tense and nervous when doing homework,” scored on a 5-point scale, with 1 indicating “strongly disagree” and 5 indicating “strongly agree.” Mean scores across these four items represented individual scores for homework anxiety; higher values signified elevated levels of anxiety related to homework completion. In the present study, the internal consistency coefficient was 0.904. The model fitness generated by confirmatory factor analysis was good: *χ^2^/df* = 0.010, RMSEA = 0.000, CFI = 1.000, TLI = 1.002, and SRMR = 0.000.

#### Statistical analysis

Data management and statistics were conducted utilizing SPSS 26.0 and Mplus 8.0. SPSS 26.0 facilitated descriptive statistics alongside correlation assessments among variables. Mplus 8.0 was used to construct structure equation modeling to first test the direct effect model between parental structure versus chaos, and homework anxiety, and based on which the mediating role of homework motivation (including autonomous motivation and controlled motivation) was further tested. It should be noted that students’ gender, age, and family socioeconomic status were controlled as covariates in testing the direct and mediated effects models. The chi-square (*χ^2^*), degrees of freedom (*df*), the root mean square of error of approximation (RMSEA), the comparative fit index (CFI), the Tucker -Lewis index (TLI), and the standardized root mean square residual (SRMR) were used to assess the model fit. In addition, a bootstrap analysis was conducted to examine significance of the mediation effect with 1,000 bootstrap samples set at 95% confidence intervals (CIs). The mediation effect is significant if the 95% CI does not contain zero.

## Results

### Common method bias

Given that all data were derived from self-reports, a common method bias test was performed using the Harman’s single factor before proceeding with the results. The results revealed that six principal component factors had eigenvalues greater than 1. The variance explained by the first factor was 29.088%, which is below the critical threshold of 40% (Zhou and Long, 2004; Chaubey et al., 2019). This finding suggests that there is no significant common method bias in the data.

### Means, standard deviations and correlations of variables

Table 1 presents the mean, standard deviation, and correlations among the variables. The correlation analysis indicated that parental structure was significantly positively correlated with autonomous motivation and significantly negatively correlated with homework anxiety; parental chaos was significantly negatively correlated with autonomous motivation and significantly positively correlated with controlled motivation and homework anxiety; autonomous motivation was significantly negatively correlated with homework anxiety; and controlled motivation was significantly positively correlated with homework anxiety. In addition, the correlation coefficients of gender with autonomous motivation as well as age with parental structure and chaos were significant, thus gender and age were included as control variables in the subsequent analyses in order to exclude their influence on the study results.

**Table 1:** Means (M), standard deviations (SD), and correlations between variables.

	*M*	*SD*	1	2	3	4	5	6
Gender	1.500	0.500	–					
Age	10.560	1.735	0.021	–				
PS	3.913	0.791	−0.051	−0.160^***^	–			
PC	2.640	0.992	−0.042	0.061^*^	−0.308^***^	–		
AM	3.726	0.757	0.006	−0.020	0.365^***^	−0.208^***^	–	
CM	3.148	0.829	−0.109^***^	−0.048	0.013	0.177^***^	0.007	–
HA	2.348	0.989	−0.044	0.021	−0.183^***^	0.339^***^	−0.350^***^	0.390^***^

PS, parental structure; PC, parental chaos; AM, autonomous motivation; CM, controlled motivation; HA, homework anxiety. For gender, 1 = male, 2 = female. ^*^p < 0.05, ^**^p < 0.01, ^***^p < 0.001.

### Direct effect model

**Figure 2:**
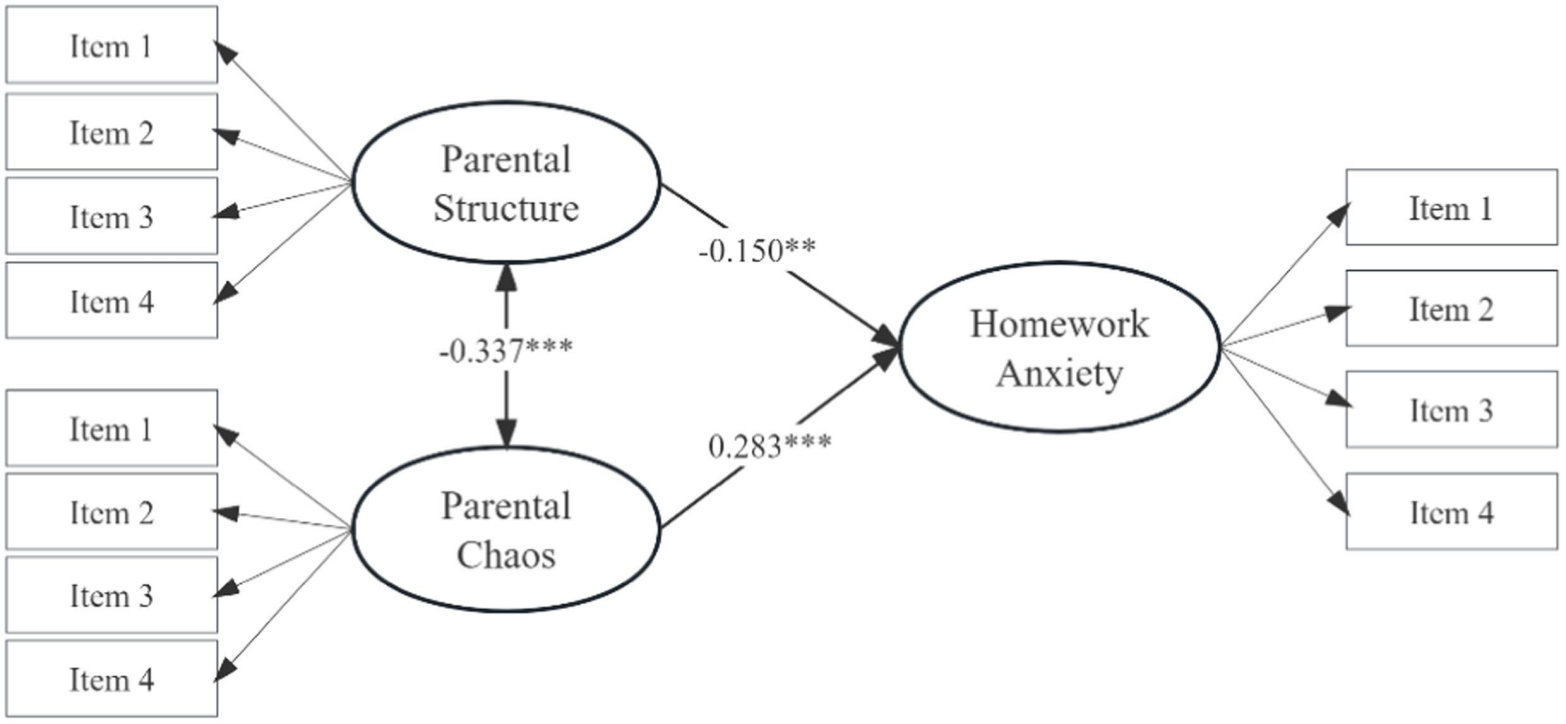
The direct effect model. *p < 0.05, **p < 0.01, ***p < 0.001, the factor loadings of items 1–4 for parental structure, chaos, and homework anxiety range from 0.569–0.877, 0.554–0.798, and 0.691–0.951, respectively. Gender, age, and socio-economic status are used as control variables, but are not shown in the figure for brevity.

To examine the effects of the parental structure and chaos on homework anxiety, a direct effect model was established with parental structure and chaos as the independent variables and homework anxiety as the dependent variable, as shown in Figure 2. In this model, parental structure, chaos and homework anxiety were latent variables (each with 4 entries); gender, age, and socioeconomic status were used as covariates. The model demonstrated good fit indices: *χ^2^/df* = 2.895, CFI = 0.970, TLI = 0.963, RMSEA [90% CI] = 0.041 [0.036, 0.047], and SRMR = 0.059. Parental structure was a negative predictor of homework anxiety (*β* = −0.150, *p* = 0.001); parental chaos was a negative predictor of homework anxiety (*β* = 0.283, *p* < 0.001). Parental structure and chaos explained 6.6 and 6.9% of homework anxiety, respectively. In addition, parameter estimation of the original sample construct of 1,000 random samples with put-backs revealed 95% confidence intervals of [−0.219, −0.065] for the predictive effect of parental structure on homework anxiety, and [0.200, 0.387] for the predictive effect of parental chaos on homework anxiety, neither of which contained zeros, and Hypothesis 1 was supported.

### Mediating effect model

**Figure 3:**
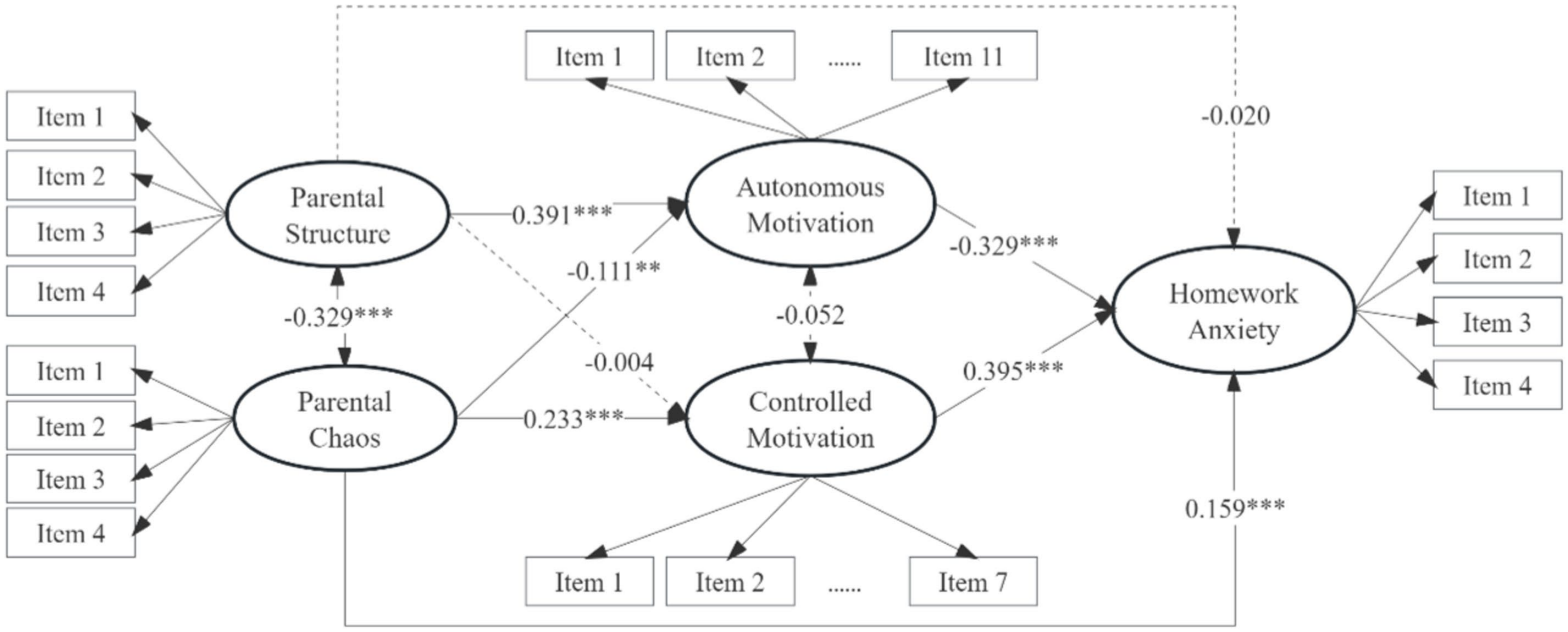
The mediating model. *p < 0.05, **p < 0.01, ***p < 0.001, factor loadings for items 1–4 for parental structure, chaos, autonomous motivation, controlled motivation, and homework anxiety ranged from 0.572–0.872, 0.561–0.791, 0.644–0.831, 0.555–0.815, and 0.699–0.941, respectively. Gender, age, and socio-economic status are used as control variables, but are not shown in the figure for brevity.

To explore the mediating role of homework motivation between the parental structure, chaos and homework anxiety, a mediating effect model was constructed by incorporating homework motivation (comprising autonomous and controlled motivation) into the direct effect model, as illustrated in Figure 3. In this model, parental structure and chaos, autonomous motivation, controlled motivation, and homework anxiety were latent variables (each with 4, 4, 11, 7, and 4 items). Gender, age, and socioeconomic status were taken as covariates. The model fit was good: *χ^2^/df* = 3.929, CFI = 0.928, TLI = 0.916, RMSEA [90% CI] = 0.051 [0.049, 0.054], and SRMR = 0.061. The results indicated significant direct effects of parental structure on autonomous motivation, parental chaos on autonomous motivation and controlled motivation, and both forms of motivation on homework anxiety. Autonomous motivation was found to mediate the relationships between parental structure and homework anxiety, as well as between parental chaos and homework anxiety. Controlled motivation mediated the relationship between parental chaos and homework anxiety. Detailed analysis of these mediating effects is presented in Table 2.

**Table 2:** Bootstrapping results of the mediating effect.

Pathway	Est	S.E.	*p*	95% CIs
PS – AM – HA	−0.129***	0.021	<0.001	[−0.145, −0.004]
PC – AM – HA	0.036**	0.027	0.006	[0.007, 0.109]
PS – CM – HA	−0.002	0.013	0.919	[0.008, 0.144]
PC – CM – HA	0.092***	0.021	<0.001	[0.001, 0.068]

PS, parental structure; PC, parental chaos; AM, autonomous motivation; CM, controlled motivation; HA, homework anxiety, *p < 0.05, **p < 0.01, ***p < 0.001.

## Discussion

This study examined the influence of parental structure and chaos on elementary school students’ homework anxiety and its mechanism of action, further deepening the understanding of the importance of establishing parental structure. The present study revealed a close link between parental structure and chaos with homework anxiety. The findings suggest that parental structure could be negatively associated with homework anxiety indirectly through homework autonomous motivation; parental chaos could be positively associated with homework anxiety indirectly through homework autonomous and controlled motivation.

### The influence of parental structure and chaos on homework anxiety

The findings confirm that parental structure can mitigate homework anxiety, whereas parental chaos tends to exacerbate it among elementary school students, reflecting the positive effects of parental structure, which aligns with previous research (Dumont et al., 2014; Farkas and Grolnick, 2010; Griffith and Grolnick, 2014; Grolnick et al., 2014; Grolnick et al., 2015). Given that elementary education represents an initial phase where students must navigate complex academic tasks amidst competitive pressures for advancement (Chen, 2010; Liu et al., 2018), effectively managing these challenges is crucial for maintaining psychological well-being (Sun et al., 2021). Therefore, parents are encouraged to actively establish structured environments that foster academic development while alleviating associated anxieties—ultimately promoting holistic development. Research indicates that parents who cultivate a structured environment enable their children to develop control over academically relevant outcomes which enhances mastery motivation and self-efficacy (Flamm and Grolnick, 2013; Skinner, 1995; Skinner et al., 2008), thereby facilitating academic development. When parents delineate clear rules regarding homework completion expectations alongside necessary guidance about behavioral consequences related to those rules they enhance children’s sense of agency over educational outcomes. This empowerment sustains motivational levels towards achieving learning objectives while fostering positive emotional experiences whilst mitigating negative ones. Furthermore, establishing such structures implies shared responsibilities within family activities which can stimulate children’s intrinsic motivation as well as accountability—thereby improving efficiency in completing assignments and reducing resultant anxieties. Conversely, existing studies have identified associations between parental chaos and increased problematic behaviors in children (e.g., Crespo et al., 2019; Hardaway et al., 2012), adversely affecting their academic trajectories. High levels of chaos may overwhelm parents’ capacity for self-regulation leading them toward maladaptive parenting styles when addressing noncompliance from their children—a dynamic detrimental to educational development (Geeraerts et al., 2021). Consequently, parental chaos complicates effective management of undesirable behaviors during assignment completion thus amplifying negative experiences associated with academics among students. Hence it becomes evident that fostering parental structure rather than succumbing to chaos plays an instrumental role in alleviating children’s adverse emotional responses linked with homework tasks.

### The mediating role of homework motivation

The results showed that parental structure can alleviate homework anxiety by enhancing children’s autonomous motivation, whereas parental chaos may exacerbate homework anxiety among elementary school students by increasing their controlled motivation and diminishing their autonomous motivation. The supportive behaviors exhibited by parents—such as establishing and enforcing rules—can positively convey educational expectations, thereby fostering confidence in children. Rather than perceiving parental guidance as pressure or cold detachment (Greenfield et al., 2006), children may interpret such actions as manifestations of parental love and concern (Chao and Kaeochinda, 2010). Existing research has demonstrated that the establishment of a structured parenting environment stimulates students’ learning motivation and encourages the development of initiative, autonomy, and self-confidence (Rogers et al., 2009). This sense of autonomy and self-assurance further enables students to experience more positive emotions while mitigating negative feelings during challenging academic situations. Students tend to report heightened enjoyment when they feel confident in their ability to complete assignments successfully; conversely, low self-efficacy regarding homework completion is associated with increased negative emotions such as anxiety (Putwain et al., 2013; Luo et al., 2016). In contrast, students exposed to high levels of parental chaos often struggle to connect their actions with outcomes; they lack intention, purposefulness, and realistic expectations concerning the results of their efforts (Deci and Ryan, 2012). Consequently, these students engage in homework not for its intrinsic value but due to external rewards or constraints. This lack of perceived control over the homework process leads them to experience elevated levels of anxiety related to academic tasks (Pekrun, 2006; Pekrun et al., 2007). In summary, greater parental structure correlates with enhanced autonomous motivation among students while concurrently reducing homework anxiety; conversely, increased parental chaos diminishes autonomous motivation while heightening controlled motivation and exacerbating homework-related stress.

### Implications and limitations

This study offers both theoretical insights and practical applications. First, it provides educators with valuable strategies for alleviating elementary school students’ homework anxiety through an exploration into how parental structure versus chaos influences this phenomenon. By addressing gaps in previous research on parent–child interactions within family education contexts at a deeper level than before—particularly highlighting the predictive role played by structured parenting environments—it suggests that clear rule-setting aligned with children’s capabilities can foster positive emotional experiences during homework completion. Such practices are essential for minimizing potential risks associated with unfavorable factors while equipping children for future academic challenges. Second, this research delivers constructive recommendations on creating well-structured parenting environments aimed at promoting positive cycles within parent–child interactions. By validating the effects exerted by both structured versus chaotic parenting on elementary school pupils’ experiences regarding homework anxiety—and elucidating underlying mediating mechanisms—the study enriches existing theories surrounding academic emotions alongside those pertaining specifically to parenting strategies. Parents should consciously adapt their approaches through discussions about specific processes involved in completing homework: providing explicit expectations along with detailed guidance will help establish orderly conditions conducive for effective task engagement whilst simultaneously nurturing children’s autonomy and confidence throughout daily academic endeavors. Third, this study emphasizes the important role of students’ autonomous motivation in reducing academic anxiety. It reminds us that parents and educators can enhance students’ autonomy by providing diverse learning resources and opportunities for self-choice, and encouraging students to participate in the decision-making process.

Despite its implications, this study has certain limitations that warrant further exploration in future research to advance understanding within this field. Firstly, regarding the sample population—due to the cognitive limitations inherent among elementary school students—the participants were primarily drawn from grades four through six. Research indicates that fourth grade often represents a pivotal transition point where cognitive abilities significantly improve, enabling more accurate self-expression (Breit et al., 2021). However, perceptions of parental structure or chaos may vary across different grade levels; thus, caution is advised when generalizing these findings beyond this specific group. Future studies should include comparisons across various elementary grades to validate and deepen our comprehension of the relationships between these variables as well as their underlying mechanisms. Secondly, given its cross-sectional design, this study elucidates only correlational relationships without addressing causal pathways. Subsequent research could benefit from intervention-based experimental designs aimed at assisting parents in establishing structured environments while assessing whether such interventions influence students’ homework anxiety—thereby clarifying causal links between these factors.

## Data Availability

The original contributions presented in the study are included in the article/supplementary material, further inquiries can be directed to the corresponding author.
